# Techno-economic dataset for energy market and capacity payment co-optimization in the Dominican Republicʼs power market

**DOI:** 10.1016/j.dib.2024.111225

**Published:** 2024-12-12

**Authors:** René Báez-Santana, Miguel Aybar-Mejía, Máximo A. Domínguez-Garabitos, Víctor S. Ocaña-Guevara

**Affiliations:** a Área de Ciencias Básicas, Instituto Tecnológico de Santo Domingo, 49 Los Próceres Avenue, Santo Domingo 10602, Dominican Republic; b Área de Ingeniería, Instituto Tecnológico de Santo Domingo, 49 Los Próceres Avenue, Santo Domingo 10602, Dominican Republic; c Facultad de Ingeniería Mecánica e Industrial, Universidad Central “Marta Abreu” de Las Villas (UCLV), Santa Clara, Cuba

**Keywords:** Renewable energy, Energy policy, Optimization modeling, Capacity credits

## Abstract

The electric power industry has an impact on fossil fuel consumption, which must be considered in decarbonization strategies. Energy systems optimization modelling can be applied to evaluate policy scenarios in the power sector to accelerate energy transitions. These modelling tools need data to simulate different scenarios in the power system to clarify the design of energy policies. For this reason, collecting and processing technical and economic data is needed to guarantee quality input for the modelling tools. This article presents a dataset for an optimization model of the generation mix and the energy demand in the power system of the Dominican Republic to determine the capacity value of variable renewable energy (VRE), i.e., wind and solar, that can serve as an incentive for these technologies. While the data corresponds to the Dominican Republic's power system, the method of collecting and processing data can be implemented in other countries. The data collected is an open-access database of the independent system operator, the power sector regulator, and utilities, as well as websites and databases of international organizations.

## Nomenclature

AF: Availability Factor
CCGT: Combined cycle gas turbine
CNE: National Energy Commission
FOR: Forced outage rate
MEM: Ministry of Energy and Mines
NERC: North American Electric Reliability Corporation
OC: Dominican independent system operator
SIE: Superintendence of Electricity
VRE: Variable renewable energy

Specifications Table

**Table:** 

Subject	Energy.	
Specific subject area	Energy policy and renewable energy incentive.	
Data format	Processed.	
	Minimal adjustments may be necessary before modelling.	
Type of data	Table (.xlsx format).	
Data collection	To collect data, information regarding power generation and variable cost was requested to the independent system operator of the Dominican Republic (Organismo Coordinador). This information was provided as raw data in an Excel file. The rest of the information was obtained from the official websites of the regulatory entity of the power sector - *Superintendencia de Electricidad* (SIE) *-*, the North American Electric Reliability Corporation (NERC) and the *Comisión Nacional de Energía* (CNE). Other general information, such as the regions and provinces in the Dominican Republic was gathered from trusted websites. All the information was put together into a single Excel file as raw data to prepare it for processing. MS Excel was considered for processing the data, however, using Python and the *Pandas* and *datetime* libraries was determined to be the optimal choice for this purpose. Missing values were treated according to the following criteria: Null values for power generation were filled with zero (0) because, according to the source (Organismo Coordinador), null values mean that no power was generated by the technology in question, hence the data is not missing. In the case of null values for variable costs in some periods, these were imputed with the mean of the variable costs of the technology and primary energy source, as having this information is important for modeling the dispatch of all generation technologies. The reason for the missing values of variable costs is that the independent system operator does not publish the variable costs of power plants until their commercial date of entry.	
Data source location	Data sources:	References
	1. Organismo Coordinador del Sistema Eléctrico Nacional Interconectado (OC)	[1,2]
	2. Superintendencia de Electricidad (SIE)	[3,4]
	3. North American Electric Reliability Corporation (NERC)	[5,6]
	4. Comisión Nacional de Energía (CNE)	[7]
Data accessibility	Repository name: Mendeley Data. “Technical and economic data for energy market and capacity payment optimisation in the Dominican Republicʼs power market”. Data identification number: 10.17632/5xss424m4p.2. Direct URL to data: https://data.mendeley.com/datasets/5xss424m4p/2	
Related research article	None.	

### 1 Value of the Data

• This data can be used for modelling the energy market and the capacity payment in the Dominican Republicʼs power market to assess different scenarios of VRE integration, and its impact on the security of supply. Hence, it is useful for regulators or decision-makers in energy policies and system operators for developing incentives based on the contribution of VRE to the power system's adequacy, by determining the capacity value of VRE (by region or by province), within the Dominican Republic's power market.
• The dataset can be reused by other researchers to gain insights from the Dominican Republic's power market as it is presented as a single database where technical and economic information merge to analyze how the energy market can be affected by the capacity payments and the firm power of the generation mix. By integrating data from multiple sources, the dataset makes the work of collecting technical and economic data easier for analysts and researchers.
• The dataset can serve as reference for researchers that work on developing energy markets and capacity payment optimization models with open data in countries or regions with similar characteristics to the Dominican power system.
• The dataset is useful for research in topics of regulation of the power sector that focuses on exploring and benchmarking methodologies that calculate the capacity value of VRE generation, considering the effect on the dispatch of thermal power plants.

### 2 Background

The interest in modeling energy market and capacity payments is what motivates the compilation of this dataset. This modeling requires specific data that, although it may be available from different sources, carries copious amount of work to gather and merge into one dataset, which poses a limitation for this kind of modelling.

Another significant dataset dedicated to the energy industry in the Dominican Republic, is the Techno-economic dataset and assumptions for long-term energy systems modelling in the Dominican Republic (2024–2050) [8]. However, this dataset compiles data from the land transport sector and the electric power sector. Also, this exercise is performed to support the energy planning policies of the Dominican Republic in the long term, which is not a data suitable for modelling the day-to-day operation of power plants.

To tackle these limitations, the dataset introduced in this paper gathers the data and the granularity of the data that is required to model the Dominican energy market and the capacity payments. The data collected relates to both energy and capacity concepts, e.g. hourly variable costs and capacity payment price, respectively. Key measures covered in this dataset include VRE power generation, thermal power plants generation, among other data, with sufficient granularity, i.e. in an hourly fashion.

This dataset may encourage energy regulators, energy policy makers and utilities to analyze methodologies that require energy market and capacity payment co-optimization in the Dominican Republic, such as establishing new capacity remuneration mechanisms and determining the impact of scarcity pricing in the power market, among other scenarios that require this type of information.

### 3 Data Description

This document presents a dataset used in an optimization model that calculates the firm power and energy dispatch thermal conventional generation in the Dominican Republic power system, considering the variable renewable generation. This work seeks to support VRE integration through an incentive that is based on the capacity value of these technologies. The data can be used in any optimization tool and can be adapted or reused to other analysis and planning tools. This dataset is a collection of information taken from various sources such as international and national organizations linked to energy policies and the power market in the Dominican Republic. The sources include technical reports and databases from these organizations.

A file is provided in the repository [9]. The file is an Excel workbook named “Technical and economic data for energy market and capacity payment optimization in the Dominican Republic's power market”, which contains the technical and economic data and assumptions for modelling the energy market and capacity payment in the Dominican Republic for 2023, that can help determine the contribution of VRE to system adequacy (see Table 1).

**Table 1:** Dataset content of the file “Technical and economic data for energy market and capacity payment optimization in the Dominican Republic's power market”.

CONTENTS	
1. Sets	
1.1	Regions
1.2	Provinces
1.3	Year
1.4	Day of the month
1.5	Daily periods
1.6	Yearly periods
1.7	Technologies
1.8	Primary energy source
2. Parameters	
2.1	Capacity payment
2.2	Scarcity price
2.3	Net effective power
2.4	Forced outage rate
2.5	Availability factor
2.6	Commercial generation and hydro
2.7	VRE generation by region
2.8	Variable costs
3. Additional data	
3.1	Total demand
3.2	Residual demand
3.3	Non-commercial generation
3.4	Hydro generation
3.5	VRE generation by province
3.6	DOP-USD rate

The first sheet of the workbook, Technical and economic data for the energy market and capacity payment optimization in the Dominican Republicʼs power market” shows a summary of the content, which is divided into three sections: Sets, Parameters, and Additional data (Table 1).

The *Sets* section presents the data used to define indices for variables and parameters. It defines the model's structure by grouping related elements. The *Parameter* section shows the data that feeds the model. In the *Additional data* section, supplementary data used to produce the data in the Parameter section is found. Furthermore, it can be used for additional analysis along with the model results. The dataset of these sections is described below using the structure shown in Table 1.

#### 3.1 Sets

This section provides the basic configuration of the model. The data establishes the regions and/or provinces where VRE generation is located, the year of operation, the day of the month, the daily periods of operation, and the yearly periods. It also shows the different types of technologies and the primary energy source.

##### 3.1.1 Regions

**Fig. 1:**
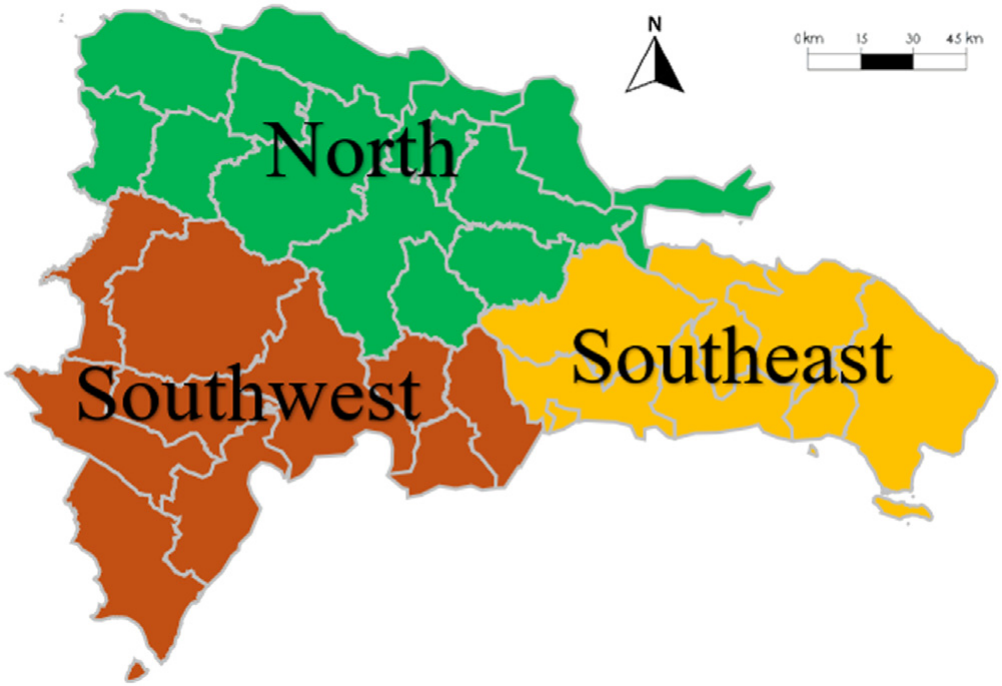
Dominican Republic's regions.

The Dominican Republic can be divided into three macro-regions: north, southeast, and southwest regions [10], as shown in Fig. 1. The location of VRE generation plants can significantly impact their production due to various factors such as resource availability and seasonal variability. This information helps by grouping VRE generation and showing the contribution to the supply and demand of each region.

##### 3.1.2 Provinces

**Fig. 2:**
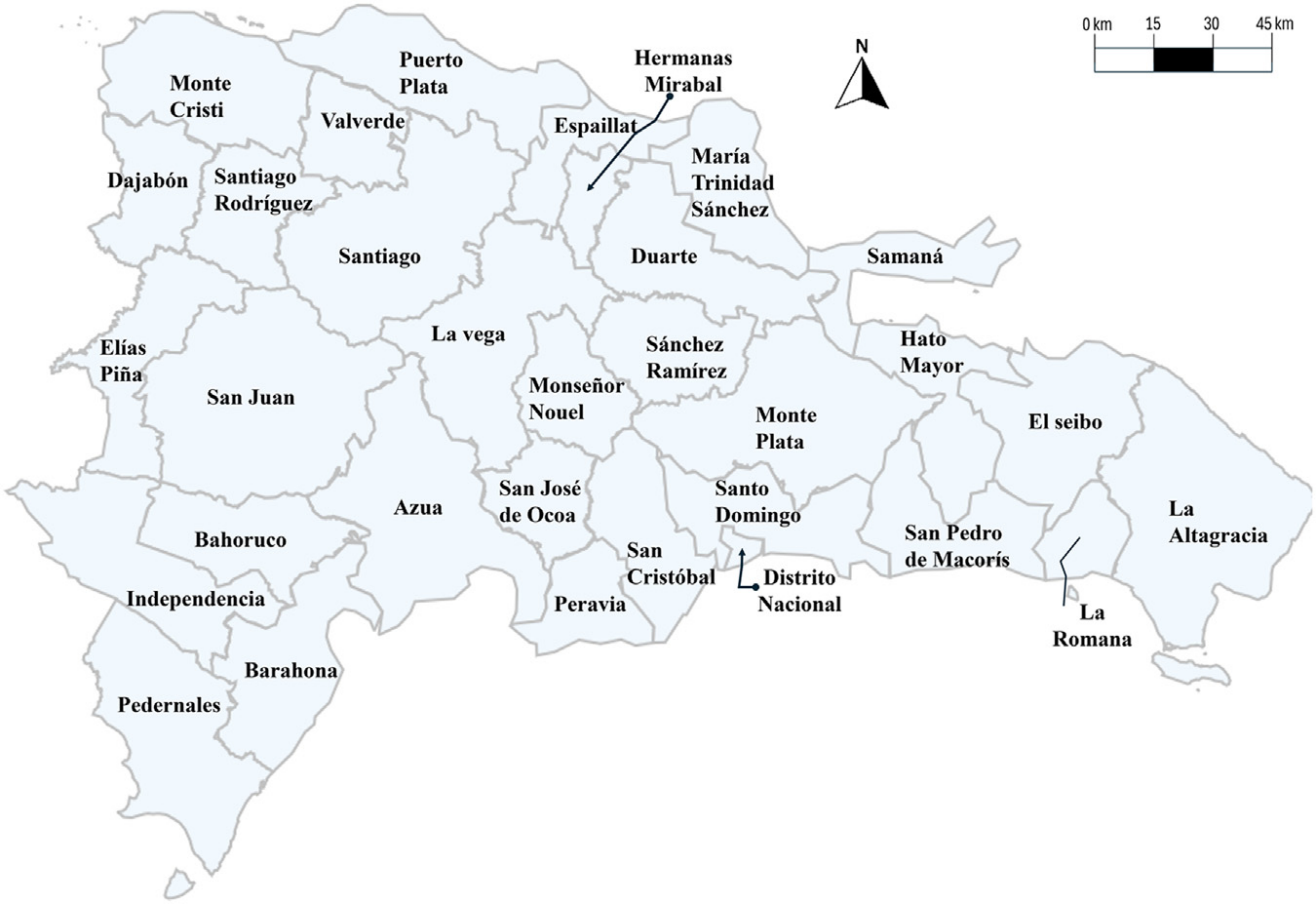
Dominican Republic physical map.

The Dominican Republic can also be divided into its provinces. It can be interesting to have the VRE generation grouped by provinces, as it shows specifically which provinces are contributing more to the development of VRE generation plants. The physical map in Fig. 2 shows all provinces in the Dominican Republic.

However, only eleven provinces are shown in the dataset, as those are the provinces where VRE projects have been developed at utility scale. These provinces are: Azua, Barahona, La Romana, María Trinidad Sánchez, Monte Cristi, Monte Plata, Peravia, Puerto Plata, San Cristóbal, San Pedro de Macorís and Santo Domingo.

##### 3.1.3 Year

Defining the modelling time window is important. In an optimization model, resources are going to be optimally allocated, and this depends on the defined period or time window. For the data presented, only one year (2023) is considered, as it is oriented towards a medium-to-long-term model suited for system firmness analysis. The capacity payment mechanism in the Dominican Republic is based on the firm power of power plants during a calendar year, hence, one year of data is sufficient to obtain reliable results from a co-optimization model of the hourly energy market and the capacity payment in the Dominican context.

##### 3.1.4 Day of the month

It defines the number of days within a month. The number of days will depend on the month in question.

##### 3.1.5 Daily periods

Daily periods refer to the hours within a day. Daily periods are important because they show how different factors change throughout the day. With an hourly resolution, the VRE generation can be appreciated, as well as the demand fluctuation and VRE generation. Furthermore, the energy dispatch that results from the model will also be given in an hourly manner for each day.

##### 3.1.6 Yearly periods

Yearly periods refer to the hours within a year (8760 h for the year 2023). Yearly periods can show how demand and VRE generation have evolved during the year while optimizing the energy dispatch for each hour during the year.

##### 3.1.7 Technologies

In the Dominican Republic, a range of power generation technologies are used to produce electricity from various sources. Traditional methods are steam turbines, combined cycle gas turbines, internal combustion engines, gas turbines, and hydropower turbines. Renewable technologies, such as wind turbines and solar photovoltaics, are also deployed all around the country. Making the distinction by technologies is critical because factors such as generation cost, reliability, and resource availability have a direct impact on energy dispatch.

##### 3.1.8 Primary energy source

Technologies generate power from different sources, including biomass and fossil fuels such as coal, fuel oil #2 and #6, natural gas, and biomass, which are burned to generate heat and drive turbines. Renewable technologies use natural resources such as wind and solar to generate electricity. Because the cost of the resource affects the generation cost, it is also important to take it into account.

#### 3.2 Parameters

This section provides the basic configuration of the model. The data establishes the regions and/or provinces where VRE generation is located, the year of operation, the day of the month, the daily periods of operation as the yearly periods. It also shows the different types of technologies and the primary energy source.

##### 3.2.1 Capacity payment

The Dominican Republic has a capacity payment as a remuneration mechanism that serves as an incentive for power generators to ensure sufficient capacity and availability to meet peak demand. The regulatory entity, *Superintendencia de Electricidad*, defines a price at which the firm power of thermal and hydropower generators is valuated [3]. The reference price of 550.71 RD$/kW-month corresponds to the month of December 2020, and it is affected by a monthly index.

The price value for the capacity payment in this section is annualized. It is calculated using Eq. (1):

(1) $$Price_{Annualized\_CP}=\frac{\frac{\sum_{i=1}^{n}Price_{i}}{n}}{\frac{\sum_{i=1}^{n}USD\_rate_{i}}{n}}*1,000\frac{kW}{MW}*n=119,774.28\;USD/MW\;$$

Where:

$\frac{\sum_{i=1}^{n}Price_{i}}{n}$: Average monthly price for the year 2023.

$\frac{\sum_{i=1}^{n}USD\_rate_{i}}{n}$: Average monthly DOP/USD rate for the year 2023.

*n*: months in a year.

##### 3.2.2 Scarcity price

The scarcity price reflects the value of electricity during periods of shortage. This part shows the scarcity price for every period in the year 2023. The scarcity price and its indexing for every year are defined by the regulatory entity as well [4].

##### 3.2.3 Net effective power

Net effective power is the maximum power that a generation plant can deliver to the grid after accounting for its consumption, as shown in (2):

(2) Neteffectivepower=Grosspoweroutput−InternalConsumption

Table 2 shows the total sum of the net effective power in the Dominican interconnected power system according to the OC [1], grouped by a combination of Technology and Primary energy sources. A code is assigned to each combination to facilitate the modelling. For VRE, the region or location is also considered along with the technology to see the net effective power of these technologies by region.

**Table 2:** Power 'plants' net effective power is achieved through technology and primary energy sources.

Technology/Location	Primary energy source	Code	Net effective power [MW]
STEAM TURBINE	BIOMASS	STTUBIOM	27.8
STEAM TURBINE	COAL	STTUCOAL	957.3
CCGT	NATURAL GAS	CCGTNGAS	762.1
CCGT	GAS AND FUEL OIL #2	CCGTGFO2	259.8
INTERNAL COMBUSTION ENGINE	GAS AND FUEL OIL #6	ICENGFO6	504.0
INTERNAL COMBUSTION ENGINE	FUEL OIL #6	ICOENFO6	498.7
GAS TURBINE	NATURAL GAS	GASTNGAS	63.4
GAS TURBINE	FUEL OIL #2	GASTUFO2	99.8
SOLAR-PV-NORTH	SUN	SOLPVNOR	126.6
SOLAR-PV-SOUTHEAST	SUN	SOLPVSEA	179.9
SOLAR-PV-SOUTHWEST	SUN	SOLPVSWE	318.6
WIND-TURBINE-NORTH	WIND	WINDTNOR	199.8
WIND-TURBINE-SOUTHWEST	WIND	WINDTSWE	217.3

**Fig. 3:**
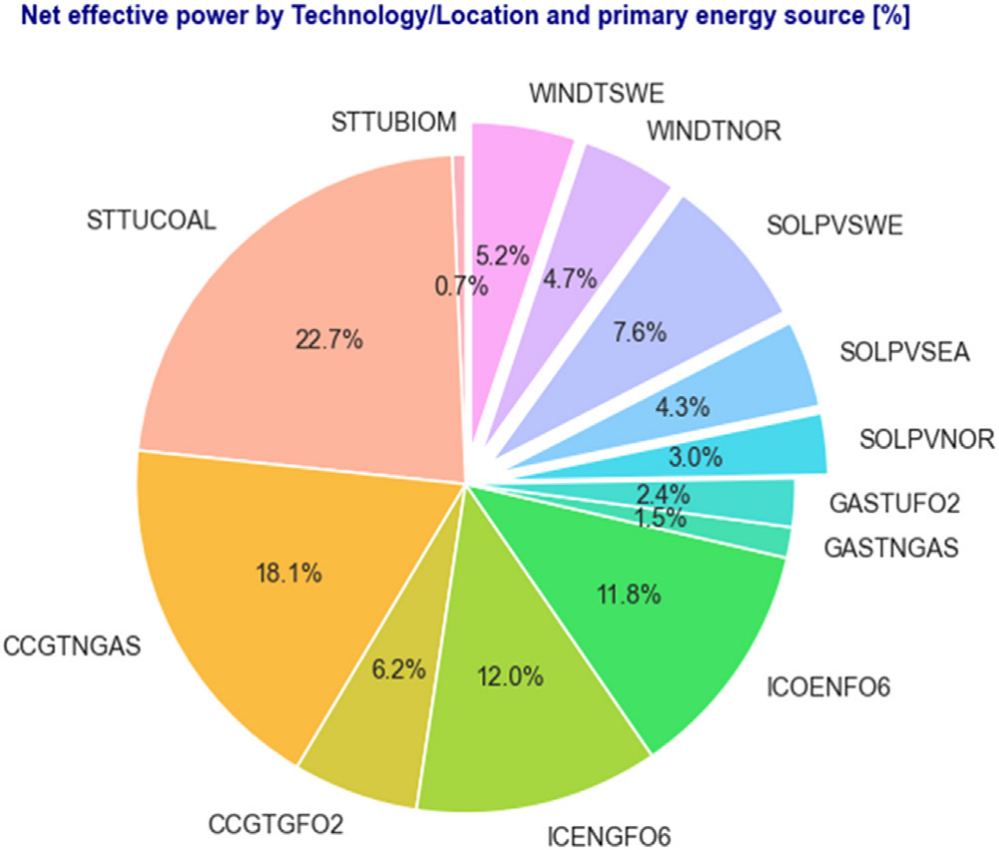
Percent of total net effective power according to the code assigned in Table 2.

Fig. 3 illustrates the share of the net effective power that VRE power plants have by location and against thermal and fossil fuel power plants. For example, Solar PV located in the southwest region (SOLPVSWE) represents the largest share of VRE power plants.

**Fig. 4:**
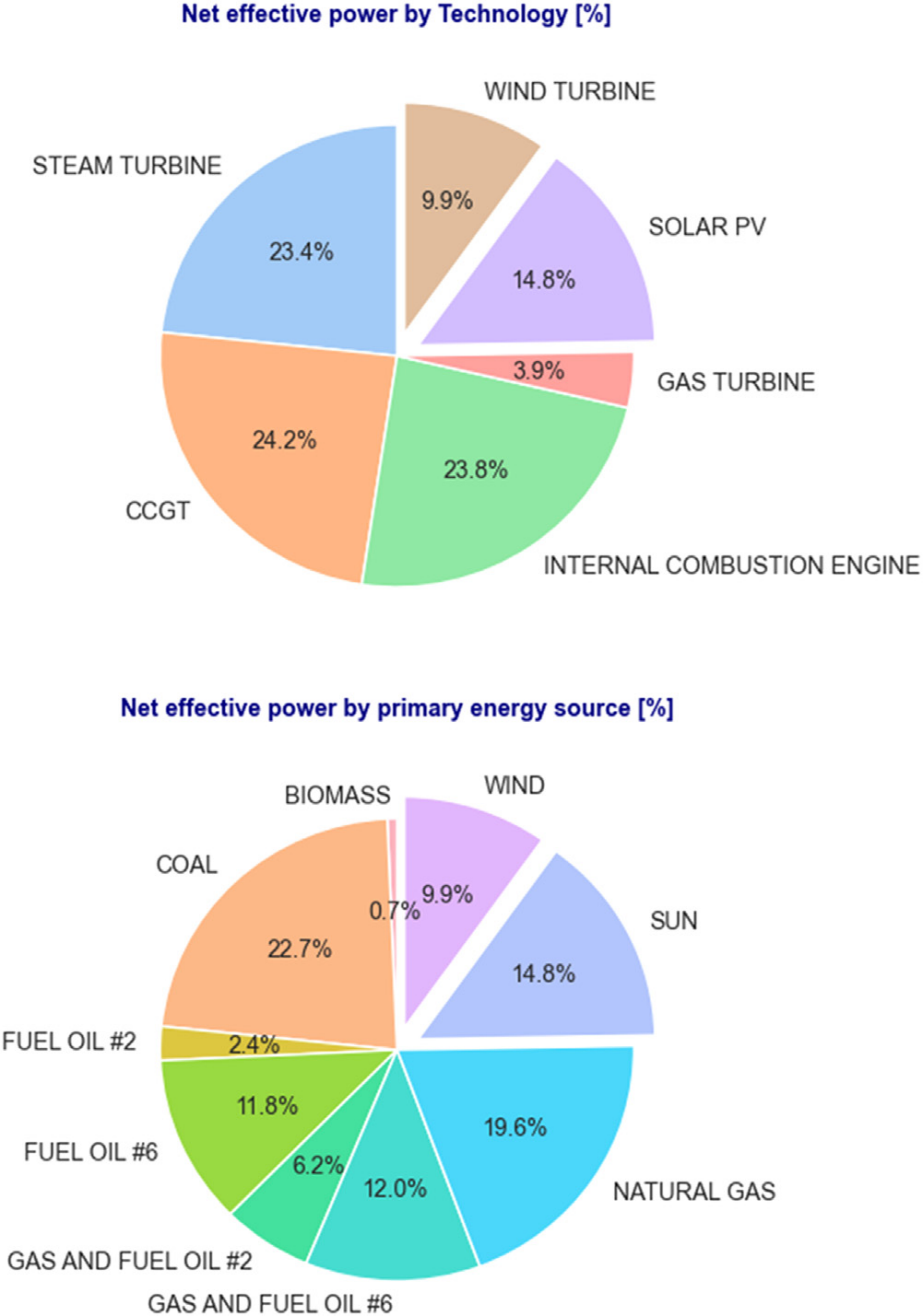
Percent of total net effective power by technology and primary source of energy.

Fig. 4, complements the previous graph by showing the distribution as a percentage of the total net effective power first by technology and then by primary energy source.

##### 3.2.4 Forced outage rate

The forced outage rate (FOR) measures the reliability and performance of power plants (see Table 3 [5]), such as turbines and engines. It represents the percentage of time that a power plant is unexpectedly out of service due to failure or other issues that were not planned. According to NERC [6], FOR is calculated with formula (3):

(3) $$FOR=\frac{FOH}{FOH+SH+Synchronous\;Condensing\;Hours+Pumping\;Hours}*100\%$$

**Table 3:** Power plants' forced outage rate by technology and primary energy source.

Technology	Primary energy source	Code	Forced outage rate
STEAM TURBINE	BIOMASS	STTUBIOM	12.41 %
STEAM TURBINE	COAL	STTUCOAL	12.41 %
CCGT	NATURAL GAS	CCGTNGAS	6.24 %
CCGT	GAS AND FUEL OIL #2	CCGTGFO2	6.24 %
INTERNAL COMBUSTION ENGINE	GAS AND FUEL OIL #6	ICENGFO6	12.07 %
INTERNAL COMBUSTION ENGINE	FUEL OIL #6	ICOENFO6	12.07 %
GAS TURBINE	NATURAL GAS	GASTNGAS	33.76 %
GAS TURBINE	FUEL OIL #2	GASTUFO2	33.76 %

Where:

**FOH**: Forced Outage Hours.

**SH**: Service Hours.

##### 3.2.5 Availability factor

The availability factor (AF) measures the proportion of time that a generating unit or system is available to produce power when it is required, excluding times when it is out of service for scheduled maintenance (see Table 4). NERC establishes formula (4) to calculate the AF [6]:

(4) $$AF=\frac{AH}{PH}*100\%$$

**Table 4:** Power ‘plants’ availability factor by technology and primary energy source.

Technology	Primary energy source	Code	Availability factor [%]
STEAM TURBINE	BIOMASS	STTUBIOM	75.01 %
STEAM TURBINE	COAL	STTUCOAL	75.01 %
CCGT	NATURAL GAS	CCGTNGAS	84.89 %
CCGT	GAS AND FUEL OIL #2	CCGTGFO2	84.89 %
INTERNAL COMBUSTION ENGINE	GAS AND FUEL OIL #6	ICENGFO6	92.19 %
INTERNAL COMBUSTION ENGINE	FUEL OIL #6	ICOENFO6	92.19 %
GAS TURBINE	NATURAL GAS	GASTNGAS	87.82 %
GAS TURBINE	FUEL OIL #2	GASTUFO2	87.82 %

Where:

**AH**: Available Hours.

**PH**: Period Hours.

##### 3.2.6 Commercial generation minus hydro generation

This section presents a vector that shows the energy produced hourly only by thermal power plants that are commercially available, i.e., power plants that are ready to be dispatched by the system operator, and the energy produced by VRE. This is calculated by deducting the non-commercial generation and hydro generation from the system's total demand. This information is shown in the following format:

The *Date* column shows the date in the format YYYY-MM-DD, and the number of days within a month is given by the *Day* column. The *Period* column presents the period (hour) for each day, while the *Yearly Period* column displays the period (hour) for the year. Finally, *Commercial generation minus hydro [MW]* represents the value of the variable of interest described in this section.

##### 3.2.7 VRE generation by region

This section presents the energy produced hourly only by VRE power plants and the region where they are located. Table 6 shows the format of this information.

*Date* exhibits the date in format YYYY-MM-DD, while *Technology* and *Region* show the type of VRE Technology (solar or wind) and the region where it is located, respectively. The Day column gives us the number of days within a month. As in Table 5, Table 6 the *Period* column presents the period (hour) for each day, while the *Yearly Period* column displays the period (hour) for the year. Finally, *Generation [MWh]* is the value of the variable of interest described in this section.

**Table 5:** Table format for the commercial generation minus hydro generation.

Date	Day	Period	Yearly Period	Commercial generation minus hydro [MWh]
2023–01–01 00:00:00	1	1	1	1752.065243
2023–01–01 00:00:00	1	2	2	1769.710706
2023–01–01 00:00:00	1	3	3	1752.825685
2023–01–01 00:00:00	1	4	4	1741.944733
2023–01–01 00:00:00	1	5	5	1754.740004
2023–01–01 00:00:00	1	6	6	1718.516775
2023–01–01 00:00:00	1	7	7	1715.893235
2023–01–01 00:00:00	1	8	8	1627.177957
2023–01–01 00:00:00	1	9	9	1638.166481
2023–01–01 00:00:00	1	10	10	1678.133478
…	…	…	…	…

**Table 6:** Table format for the VRE generation by region.

Date	Technology	Region	Day	Period	Yearly period	Generation [MWh]
2023-01-01 00:00:00	SOLAR PV	NORTH	1	1	1	0
2023-01-01 00:00:00	SOLAR PV	NORTH	1	2	2	0
2023-01-01 00:00:00	SOLAR PV	NORTH	1	3	3	0
2023-01-01 00:00:00	SOLAR PV	NORTH	1	4	4	0
2023-01-01 00:00:00	SOLAR PV	NORTH	1	5	5	0
2023-01-01 00:00:00	SOLAR PV	NORTH	1	6	6	0
2023-01-01 00:00:00	SOLAR PV	NORTH	1	7	7	0
2023-01-01 00:00:00	SOLAR PV	NORTH	1	8	8	0.302045294
2023-01-01 00:00:00	SOLAR PV	NORTH	1	9	9	5.860910376
2023-01-01 00:00:00	SOLAR PV	NORTH	1	10	10	6.719270504
…	…	…	…	…	…	…

##### 3.2.8 Variable costs

The variable costs of power plants refer to the expenses that change based on the amount of electricity generated. In the context of the Dominican wholesale electricity market, variable costs are composed of fuel costs and non-fuel costs.

The variable costs of thermal power plants are classified by technology and primary energy source in this section. Variable costs are published daily by the OC in the day-ahead market report [2], and to obtain it, it was necessary to extract the information from all daily reports. It is important to note that the variable cost in the daily report is used for every period of the day. The last column of this table shows the DOP-USD rate used to convert the variable cost from Dominican pesos (DOP) to United States Dollars (USD).

##### 3.2.9 Additional data

This section includes supplementary data used to generate some of the data provided in the Parameters section. This data can also be used for further analysis and modelling to study different demand and generation scenarios so that decision-makers have essential information for the development of energy policies. The additional data in this section includes the total demand, residual demand, non-commercial generation, hydro generation, and VRE generation by province.

##### 3.2.10 Total demand

The demand for the Dominican power system is presented in this section. This value comes from the sum of all commercial metering of all power plants connected to the system, which means that losses are also included, and it is displayed for every period of the year. This data is obtained from the OC.

##### 3.2.11 Residual demand

The residual demand accounts for the remaining demand that must be supplied after discounting VRE and non-commercial generation, i.e., thermal or fossil-fueled generation and hydroelectric generation.

**Fig. 5:**
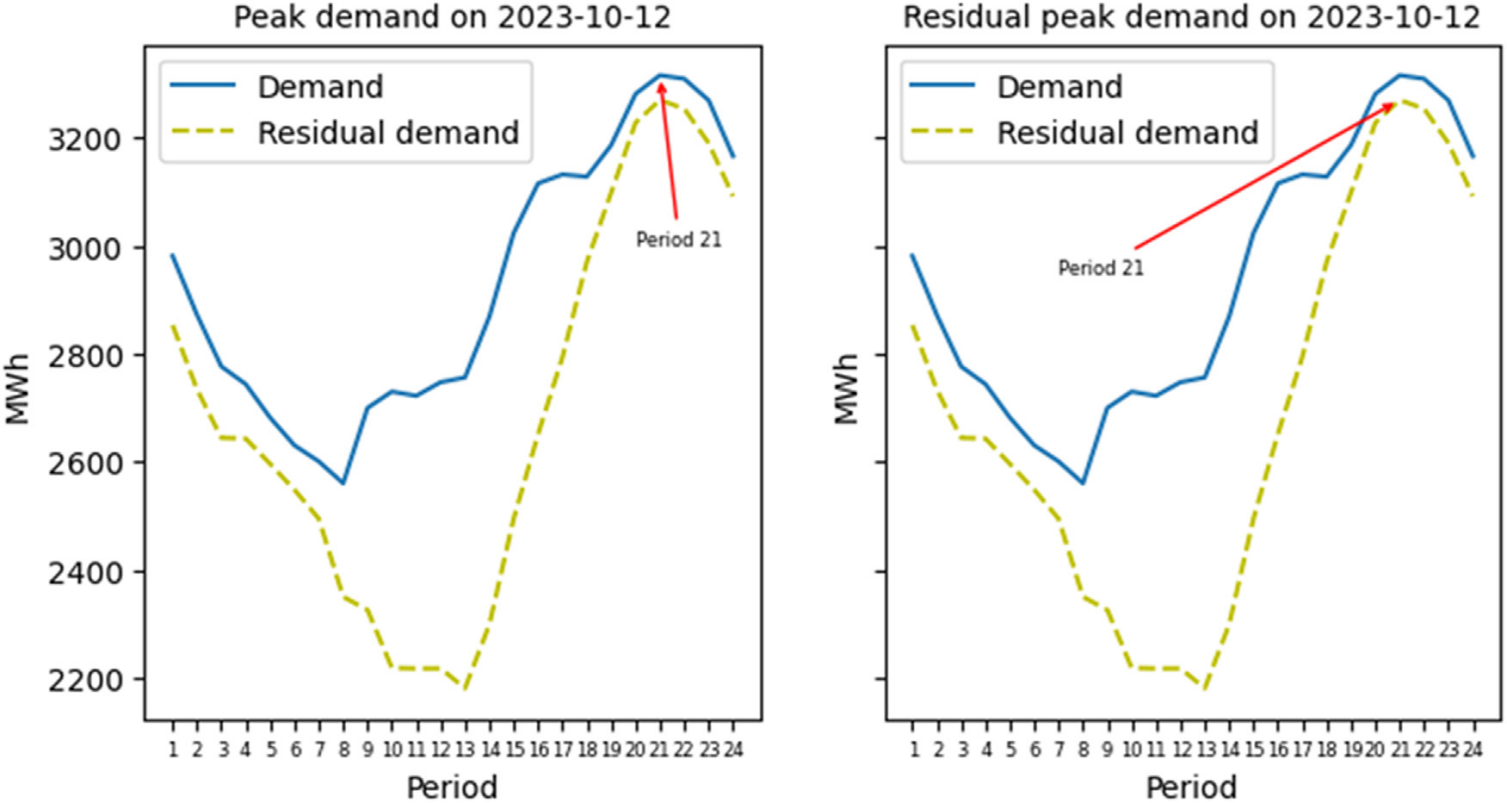
Comparison of the peak demand and the residual peak demand.

The residual demand allows to see the contribution of VRE and non-commercial generation to supplying the demand for every hour, including peak hours. Because this contribution depends on the availability of renewable resources, the residual peak demand may occur in a different period and day of the year than the total peak demand. Nevertheless, for this dataset, both the residual peak demand and the total peak demand fell in the same period and day of the year, as Fig. 5 shows.

##### 3.2.12 Non-commercial generation

This section presents a vector that shows the energy produced hourly by thermal power plants that are not commercially available, i.e., power plants that are not available to be dispatched by the system operator (see Table 7).

**Table 7:** Table format for non-commercial generation.

Date	Day	Period	Yearly Period	Non-commercial generation [MW]
2023-01-01 00:00:00	1	1	1	5.855572397
2023-01-01 00:00:00	1	2	2	0
2023-01-01 00:00:00	1	3	3	0
2023-01-01 00:00:00	1	4	4	8.751513383
2023-01-01 00:00:00	1	5	5	0
2023-01-01 00:00:00	1	6	6	3.008517194
2023-01-01 00:00:00	1	7	7	4.958834005
2023-01-01 00:00:00	1	8	8	4.67923945
2023-01-01 00:00:00	1	9	9	0
2023-01-01 00:00:00	1	10	10	0
…	…	…	…	…

##### 3.2.13 Hydroelectric generation

In the Dominican power system, two types of hydroelectric power plants can be found: run-of-the-river and reservoirs. The generation from these power plants is added up by periods for the year 2023.

##### 3.2.14 VRE generation by province

This section presents similar information to that given in Section 2.2.7, with the difference in the grouping of the data, which now groups the VRE generation by province instead of region. Table 8 shows the format of this information in a similar way to that of Table 6.

**Table 8:** Table format for the VRE generation by province.

Date	Technology	Province	Day	Period	Yearly period	Generation [MWh]
2023-01-01 00:00:00	SOLAR PV	AZUA	1	1	1	0
2023-01-01 00:00:00	SOLAR PV	AZUA	1	2	2	0
2023-01-01 00:00:00	SOLAR PV	AZUA	1	3	3	0
2023-01-01 00:00:00	SOLAR PV	AZUA	1	4	4	0
2023-01-01 00:00:00	SOLAR PV	AZUA	1	5	5	0
2023-01-01 00:00:00	SOLAR PV	AZUA	1	6	6	0
2023-01-01 00:00:00	SOLAR PV	AZUA	1	7	7	0
2023-01-01 00:00:00	SOLAR PV	AZUA	1	8	8	0
2023-01-01 00:00:00	SOLAR PV	AZUA	1	9	9	0
2023-01-01 00:00:00	SOLAR PV	AZUA	1	10	10	0
…	…	…	…	…	…	…

### 4 Experimental Design, Materials and Methods

The dataset is a collection of data from reports, websites, and databases of international organizations, such as the North American Electric Reliability Corporation (NERC), and national institutions such as independent system operator (*Organismo Coordinador del Sistema Eléctrico Nacional Interconectado* - OC), the energy policy makers (*Ministerio de Energía y Minas* – MEM and *Comisión Nacional de Energía* - CNE), and the regulatory entity (*Superintendencia de Electricidad* - SIE).

The data collected from the sources previously mentioned was merged into a single Excel File. Nevertheless, this information was not ready for analysis and could be simplified for modelling purposes. Python was used for this task, along with the *Pandas* and *datetime* libraries. The data was gathered and processed during the month of September 2024.

The data corresponding to energy generation was initially given by the power plant. It was transposed into a long format and then added up by technology and/or primary energy source in the case of thermal/fossil-fueled power plants and added up by region or province in the case of VRE generation (wind and solar). To determine the generation corresponding to each region, it was necessary to access a map available on the CNE home page [7]. A similar approach was taken regarding hydroelectric generation and non-commercial generation, where the generation of all power plants was added up according to these criteria.

Concerning variable costs of thermal power plants, a USD rate was considered for converting these costs from Dominican pesos to United States Dollars. This data was grouped by technology and primary energy source by calculating the average of the variable costs of all power plants in every technology-primary energy resource pair. Afterward, all missing values were filled with estimated imputations, which, in this case, was the average of the variable costs corresponding to the technology and primary energy source in question for the year.

### Limitations

In the case of the Dominican Republic and its institutions linked to the power sector, information is not organized on digital platforms that allow easy access to information. Additionally, when it is available, it requires pre-processing to clean, organize and build a dataset that is ready for modelling. Another limitation of the dataset is that it provides data only for a year (2023), which prevents us from evaluating how the data can change over the years.

### Ethics Statement

The authors declare that the generation of this dataset did not involve human subjects, animal experiments, or data collected from social media platforms.

### CRediT Author Statement

**René Báez-Santana:** Conceptualization, methodology, data curation, writing—original draft preparation, writing—review and editing, supervision. **Miguel Aybar-Mejía:** Conceptualization, methodology, writing—original draft preparation, writing—review and editing, supervision. **Máximo Domínguez:** Conceptualization, methodology, writing—original draft preparation, writing—review and editing, supervision. **Víctor Ocaña:** Conceptualization, methodology, writing—original draft preparation, writing—review and editing, supervision.

All authors have read and agreed to the published version of the manuscript.

## Data Availability

Mendeley DataTechnical and economic data for energy market and capacity payment optimisation in the Dominican Republic's power market (Original data).
